# The Uncertainty Assessment by the Monte Carlo Analysis of NDVI Measurements Based on Multispectral UAV Imagery

**DOI:** 10.3390/s24092696

**Published:** 2024-04-24

**Authors:** Fatemeh Khalesi, Imran Ahmed, Pasquale Daponte, Francesco Picariello, Luca De Vito, Ioan Tudosa

**Affiliations:** Department of Engineering, University of Sannio, 82100 Benevento, Italy; iahmed@unisannio.it (I.A.); daponte@unisannio.it (P.D.); fpicariello@unisannio.it (F.P.); devito@unisannio.it (L.D.V.); ioan.tudosa@unisannio.it (I.T.)

**Keywords:** vegetation index, measurement uncertainty, multispectral and hyperspectral imaging, PA, UAVs, NDVI

## Abstract

This paper proposes a workflow to assess the uncertainty of the Normalized Difference Vegetation Index (NDVI), a critical index used in precision agriculture to determine plant health. From a metrological perspective, it is crucial to evaluate the quality of vegetation indices, which are usually obtained by processing multispectral images for measuring vegetation, soil, and environmental parameters. For this reason, it is important to assess how the NVDI measurement is affected by the camera characteristics, light environmental conditions, as well as atmospheric and seasonal/weather conditions. The proposed study investigates the impact of atmospheric conditions on solar irradiation and vegetation reflection captured by a multispectral UAV camera in the red and near-infrared bands and the variation of the nominal wavelengths of the camera in these bands. Specifically, the study examines the influence of atmospheric conditions in three scenarios: dry–clear, humid–hazy, and a combination of both. Furthermore, this investigation takes into account solar irradiance variability and the signal-to-noise ratio (SNR) of the camera. Through Monte Carlo simulations, a sensitivity analysis is carried out against each of the above-mentioned uncertainty sources and their combination. The obtained results demonstrate that the main contributors to the NVDI uncertainty are the atmospheric conditions, the nominal wavelength tolerance of the camera, and the variability of the NDVI values within the considered leaf conditions (dry and fresh).

## 1. Introduction

Agriculture plays a crucial role in boosting the economy by meeting basic human needs for food and fiber. To make farming sustainable in today’s world, it is important to adopt precision agriculture (PA). PA involves using advanced data and analysis tools for precise field management, complying with several timing, application rate (i.e., fertilizers, pesticides), and spatial distribution constraints [1,2]. In other words, PA represents an innovative approach wherein farmers apply optimized inputs, including water and fertilizer, intending to improve productivity, quality, and overall yield [3]. PA is seen as a promising solution to meet the growing demand for sustainable food and energy production by optimizing and managing the external factors [1,2].

Remote sensing (RS) systems, facilitated by information and communication technologies, generate significant data characterized by high spatial, spectral, radiometric, and temporal resolutions. These attributes render such systems well suited for applications in PA. Unmanned Aerial Vehicles (UAVs) equipped with multispectral cameras designed for RS present novel prospects for ecological and agricultural applications [4,5]. These applications encompass activities such as modeling, mapping, and monitoring. UAVs, compared with satellites, have a higher resolution, a lower cost, and the possibility of conducting surveys for lower sampling periods [6]. However, using multispectral cameras for precision farming faces challenges such as limited spatial resolution compared to hyperspectral sensors, cost barriers to acquiring high-resolution hyperspectral data, and the need for precise alignment and complex data-fusion techniques when integrating data from different sensors on UAVs [7]. Furthermore, managing the complexity of processing and analyzing data from multiple spectral bands presents a significant hurdle. This complexity often requires advanced software tools and expertise in remote sensing techniques [8]. Also, ensuring accurate spectral calibration across different bands is essential, but can be challenging due to variations in sensor characteristics and environmental conditions [9]. Additionally, achieving precise geometric correction to align images with ground truth data or other spatial datasets can be difficult, particularly in dynamic environments with factors such as UAV motion and terrain variations [10,11].

Vegetation indices (VIs) obtained through UAV image analysis provide valuable insights into vegetation phenology and plant attributes associated with nitrogen levels [12], chlorophyll content [13], and plant phenotyping [14]. These indices are extracted from computer vision and image-processing algorithms that manipulate color and hyperspectral data, combining information from red–green–blue (RGB), near-infrared (NIR), far-infrared, and other cameras to enhance relevant features while mitigating environmental disturbances [15,16]. The utilization of VIs allows researchers to effectively detect, quantify, and identify anomalies in crops [14]. This capability facilitates the monitoring of crop health parameters, soil nutrient content, water availability, temperature distribution, indirect photosynthetic activity, and responding to various stressors [1,17,18,19]. The Normalized Difference Vegetation Index (NDVI) is a widely recognized and extensively employed vegetation metric derived from the NIR to red (R) reflectance [20,21]. This index has long held a prominent position as the standard methodology in RS for the comprehensive assessment of plant health, leaf area index, biomass quantification, plant productivity, and fractional vegetation cover in the agricultural industry [19,22,23].

The quality of UAV imagery data is subject to various influencing factors, including sensor characteristics, camera noise, topographic variations, lighting geometry, and meteorological conditions [24,25]. Consequently, the pixel values in the captured images at different wavelengths do not accurately reflect the surface reflectance, due to their susceptibility to these influence factors [24,25]. For this reason, to facilitate quantitative analysis in RS applications, radiometric compensation of multispectral images is essential [25,26,27]. This process involves converting pixel values into units of scene reflectance to account for atmospheric and solar conditions, ensuring accuracy in quantitative RS. Radiometric compensation for images acquired by UAV platforms is inherently challenging due to variations in imaging conditions for each capture. Therefore, an established and systematic procedure is imperative to conduct comprehensive radiometric compensation and generate multispectral images with reflectance [28,29,30].

From a methodological perspective, there remains ambiguity regarding the efficacy of measurement indices, such as the NDVI, in accurately representing the actual value of the measured quantity in PA. The assessment of uncertainties associated with these measurement indices poses an unresolved challenge. Various sources of uncertainty, encompassing atmospheric and light conditions, flight parameters (including flight altitude and image overlap), environmental variables (e.g., temperature and air humidity), sensor tilt, camera sensor limitations (e.g., image resolution), object radiation emissivity, and other factors, have the potential to significantly impact the measurement outcomes. A comprehensive understanding and effective mitigation of these sources of uncertainty are imperative for enhancing the accuracy and reliability of measurements in the context of PA [31].

However, the NVDI measurement process is a complex procedure that involves several non-linear operations. In those cases, Monte Carlo simulations are used for assessing uncertainty [32]. In [32], the authors propose a framework based on Monte Carlo simulations for sensitivity analysis to quantify the effects of influence quantities on the measurement uncertainty of a generic instrument. This framework has been applied here to NVDI measurements to quantify the effect of the uncertainty sources. The performed analysis and the obtained results could be used as a guideline for researchers who need to plan a survey for PA and want to determine the impact of sensor parameters, flight parameters, and environmental conditions on the distribution of NDVI measurements.

Two preliminary models for assessing the uncertainty associated with NDVI measurements have been previously proposed in [31,33]. While these models incorporate vegetation reflectance within the tolerance of the camera’s nominal wavelengths, with its fixed bandwidth, and consider the incident optical density (OD) captured by the camera, it is pertinent to acknowledge that practical scenarios introduce additional sources of uncertainty. These encompass atmospheric conditions in dry, humid, and mixed scenarios, uncertainty in solar irradiance, and the signal-to-noise ratio (SNR) of the cameras. This paper extends the study proposed in [33] with a sensitivity analysis of the effect of several uncertainty sources on NDVI measurement, considering the constrained bandwidth and uncertainties associated with the nominal working wavelength of the camera, incident light OD, atmospheric conditions in dry, humid, and mixed scenarios, uncertainty in solar irradiance, and the SNR of the cameras. Furthermore, it should be noted that, in practical applications, the vegetation condition is only labeled in a binary or at most a discrete number of states (e.g., fresh or dry). However, the distribution of the actual NDVI values of the observed leaves does not match such a discrete model, but it is spread as a wide range of values. As a consequence, the variability of the NDVI values within each considered state should be taken into account as an uncertainty source.

This study wants to evaluate the variability of the NDVI in specific atmospheric conditions, as a result of the considered uncertainty sources. This finds application in all cases when the whole survey is completed within the same atmospheric conditions and the NDVI values are compared within the same survey. This is the case in many applications where decisions must be made based on a single survey and ground references about the state of some parts of the crop. In this case, it is not needed to evaluate the influence of the radiometric compensation on the uncertainty. In a more complex case, when the NDVI data, coming from several surveys and eventually obtained in different atmospheric conditions are compared, it is instead essential to include the radiometric compensation. This allows obtaining NDVI values, which, for each survey, are traceable to the same references and, therefore, are mutually comparable. However, the uncertainty contribution of the compensation must be considered, as well. This latter case is left to a future study.

The subsequent sections of the paper are structured as follows: Section 2 provides an overview of relevant works about the challenges and compensation approaches related to measuring multispectral images captured by UAVs and delineates RS’s fundamentals. Additionally, it describes the proposed workflow for NDVI measurements, highlighting the primary sources of uncertainty and conducting sensitivity analysis through Monte Carlo simulations. Section 3 presents the acquired results. Assessing how the NVDI uncertainty can affect the performance of algorithms used to distinguish between healthy and unhealthy vegetation is discussed in Section 4. Finally, the concluding section serves to summarize the pertinent topics discussed in the paper while outlining future activities in the field.

## 2. Materials and Methods

### 2.1. Related Works

The paper [34] asserts that the NDVI will undeniably persist as a predominant vegetation metric. However, the efficacy of the NDVI depends on the quality of multispectral data and the accurate interpretation of the NDVI values, and it is noteworthy that no two RS images are identical. The primary challenges associated with the NDVI encompass its susceptibility to atmospheric effects, ease of saturation, and variations in sensor quality. This paper critically examines and explains these significant challenges to guide NDVI users, particularly end-users lacking in-depth RS expertise, toward a cautious utilization of NDVI data.

The study conducted by [35] claims to enhance hyperspectral imaging from unmanned aerial systems under diverse weather conditions. The research focuses on addressing two key challenges: compensating data captured by the miniaturized hyperspectral sensor to ensure accurate radiometric and spectral measurements and mitigating the impact of tilting the sensor angle relative to the solar rays on downwelling irradiance data. A method is developed to effectively alleviate tilting effects and accurately correct the downwelling irradiance data. Furthermore, the study addresses striped illumination artifacts in mapping the surface reflectance factor to the hyperspectral radiance images. This is achieved through comprehensive spectral and radiometric compensation, along with irradiance correction.

The research conducted by [36] is centered on the development and testing of a comprehensive image pre-processing workflow. This workflow is designed for the radiometric and geometric correction of UAV hyperspectral data obtained from a spectrally complex environment. The detailed procedures encompass sensor compensation, radiometric correction, automatic band alignment, mosaicking, and georeferencing. The devised workflow facilitates the efficient acquisition of hypercubes from a challenging environment with limited ground control points. Unlike conventional methods that rely on photogrammetric reconstruction for mosaicking, the proposed workflow progressively generates a mosaicked output by combining different orthogonally projected hypercubes. Techniques from diverse disciplines to present a simplified workflow effective in challenging spectrally complex environments are integrated, resulting in a radiometrically and geometrically accurate mosaicked output at very high resolution.

The paper [37] presents an integrated radiometric compensation methodology tailored to high-resolution UAV-based multispectral RS utilizing miniaturized large-array commodity Complementary Metal–Oxide–Semiconductor (CMOS) cameras. The methodology encompasses both indoor and outdoor radiometric compensation techniques. The outdoor compensation procedure addresses the correction of atmospheric path radiance and reflectance. This correction is achieved through the application of an empirical line method derived from the dark target method. The proposed integrated radiometric compensation method provides a valuable benchmark for the precise radiometric compensation of large-array CMOS multispectral cameras.

In the investigation conducted in the paper [38], a thorough examination of hyperspectral atmospheric-correction techniques is undertaken, with a particular focus on mitigating challenges associated with spectral smoothing. The study identifies and reviews three primary approaches commonly employed in atmospheric correction: scene-based empirical approaches, radiative transfer modeling approaches, and hybrid approaches. In scene-based empirical approaches, methods were developed to eliminate atmospheric effects in hyperspectral imaging for deriving relative surface reflectance spectra. The atmosphere-removal algorithm in radiative transfer modeling uses theoretical techniques to simulate atmospheric influences, extracting land surface reflectance. It models the absorptive and scattering impacts of atmospheric gases and aerosols. Hybrid approaches combine radiative modeling and empirical methods to improve surface reflectance derivation from hyperspectral imaging data. The study [38] underscores the existing need for enhancements in current atmospheric-correction algorithms. Specifically, it advocates for integrating a module to model the absorption effects of atmospheric nitrogen dioxide in the visible spectrum. This proposed augmentation elevates the accuracy and efficacy of atmospheric-correction methods.

In [39], the authors compare two deep learning methods, DeepLabV3+ and a customized Convolutional Neural Network (CNN), in the application of the NDVI for vegetation detection. The evaluation focuses on their detection performance when the training and testing datasets are originated from different geographical sites with different image resolutions. Additionally, the study proposes an object-based vegetation-detection approach that incorporates the NDVI, computer vision, and Machine Learning (ML) techniques. Upon comparing the deep learning and NDVI-ML approaches, the authors observe that the NDVI-ML method yields significantly better results than the two deep learning methods. Although this may seem surprising given the general expectation that deep learning methods outperform conventional techniques, a closer examination of the results and images indicates that these findings are reasonable from two perspectives. Firstly, the optimal performance of deep learning methods necessitates a substantial amount of training data. Without sufficient data, the performance will not be good. Secondly, for satisfactory performance, it is advantageous for the training and testing images to closely resemble each other to be suitable for use in deep learning methods.

In the paper [23], thermal, multispectral, and RGB images obtained from UAVs are used to calculate various parameters related to plant growth and water consumption. Specifically, the study claims to assess the actual canopy Transpiration (Tr), soil Evaporation (E), and EvapoTranspiration (ET) of potato plants grown under different irrigation treatments on sandy soil. The traditional method of estimating Tr and E using the Two-Source Energy Balance model-Priestley–Taylor equation (TSEB-PT) with satellite-derived Land Surface Temperature (LST) has limitations in accuracy, especially at high spatial–temporal resolutions. The paper in [23] proposes an energy-flux-modeling framework based on the TSEB-PT, leveraging high-resolution thermal and multispectral data collected by UAVs. The research, conducted during drought conditions in 2018 and 2019, recorded diurnal variations of the LST in agricultural fields. The results showed that a 1m spatial resolution produced the highest correlation for estimating Tr compared to other resolutions. This paper’s model shows how well it can accurately predict irrigation needs and differentiate between drought and heat stress impacts on crop productivity.

In the paper [40], the influence of operation parameters such as the solar zenith angle (SZA), time of day (TOD), flight altitude (FA), and the growth level of rice paddies on UAV-acquired NDVI values was comprehensively evaluated. The results indicated significant impacts of these parameters on the UAV-NDVIs, with the SZA/TOD exerting the largest influence followed by the growth level and FA. Notably, smaller SZAs yielded higher signal-to-noise ratios, reflecting more realistic growth status values. These findings are crucial for optimizing flight campaigns aimed at collecting NDVI values over rice paddy fields, providing valuable insights for PA applications.

The study [41] investigates the impact of the time of day and sky conditions on various VI derived from both active and passive optical sensors, as well as from imagery captured by a UAV. Conducted in a wheat crop in southwest Germany with varying nitrogen application treatments, measurements were taken at different solar times and under sunny versus overcast conditions. The results reveal significant differences in most VIs between paired time measurements, with the smallest variations observed between 14:00 and 16:00 h. The most stable indices over the time and sky conditions were the NIR/red edge ratio, water index, and REIP index, while simple ratios like NIR/red and NIR/green were more variable. Passive hyperspectral sensors and the active Crop Circle ACS470 sensor demonstrated the most stable measurements throughout the day and under different sky conditions. Notably, the handheld passive spectrometer showed slightly higher dependency on the time and sky conditions compared to the vehicle-based sensor. The study suggests that, with the careful selection of optimized indices, both ground-based and UAV-based sensors can provide reliable measurements across varying environmental conditions, offering valuable insights for on-farm applications in PA.

In the literature, several indexes are proposed; however, their uncertainties are not often assessed. The unique contributions of the current study, particularly from a metrological perspective, involve evaluating the impact of uncertainty sources on NDVI measurements, specifically considering the influence of atmospheric conditions under three distinct scenarios: dry–clear, humid–hazy, and mixed. These assessments take into account solar irradiance uncertainty, as well as the SNR and nominal wavelength of the camera. The study utilizes Monte Carlo analyses to comprehensively assess the effects of these uncertainty sources on NDVI measurements.

### 2.2. Remote Sensing Fundamentals

RS involves the acquisition and interpretation of information about the Earth’s surface without direct physical contact. In the context of the fundamental scenario, key aspects include the use of various sensors (e.g., optical, radar, thermal), platforms (e.g., satellites, aircraft, UAVs), technologies, and methodologies to capture data from a distance. This scenario also involves understanding atmospheric conditions, compensation processes, and geometric corrections to ensure accurate and reliable data. Furthermore, it addresses the essential principles of data interpretation, image analysis, and the extraction of meaningful information from remotely sensed data, as well as the study of spectral signatures, spatial resolutions, and temporal variations in the acquired data.

**Figure 1:**
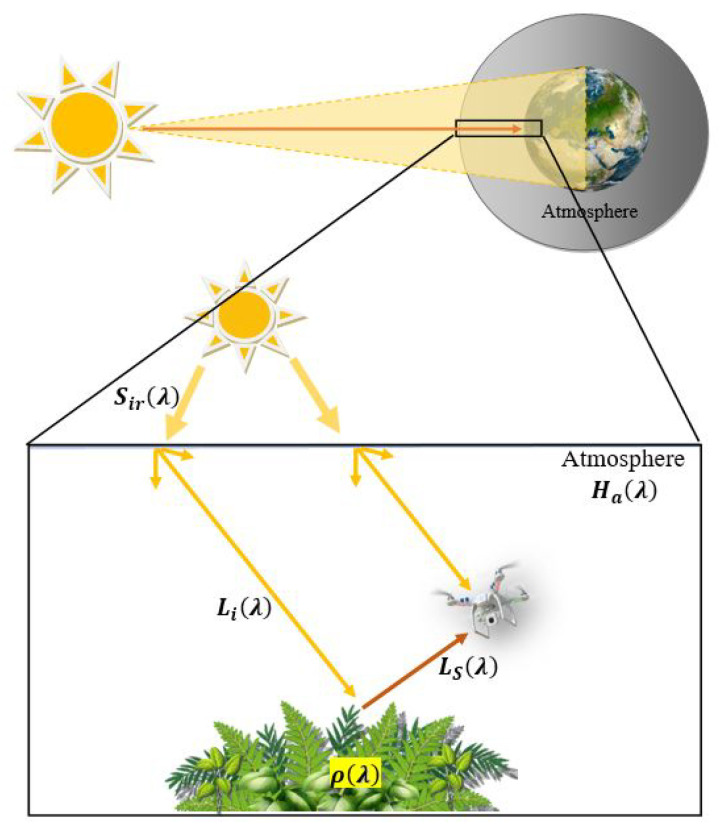
Diagram illustrating the interactions among solar radiation ($S_{ir}\left(\lambda \right)$), the Earth’s atmosphere, and the Earth’s surface. Initially, solar radiation penetrates the atmosphere and undergoes scattering and absorption by atmospheric particles and gases; $H_{a}\left(\lambda \right)$ represents the atmospheric effect response. The multispectral camera mounted on the UAV captures the reflected radiance ($L_{s}\left(\lambda \right)$), which contains valuable information about the surface properties, such as the vegetation’s features.

In the context of RS, the electromagnetic spectrum serves as a crucial domain, especially when dealing with data acquired through UAV platforms utilizing multispectral or hyperspectral imaging techniques. The interaction of solar radiation with atmospheric gases and aerosols introduces modifications to the solar irradiation spectrum before it reaches the Earth’s surface. This alteration in the spectrum impacts the radiance reflected from the Earth’s surface, a phenomenon particularly relevant when analyzing vegetation through UAV multispectral cameras. Figure 1 visually illustrates the scenario, highlighting the complex interaction between solar irradiation, atmospheric components, and the resultant reflected radiance captured by a UAV multispectral camera. Understanding and mitigating the atmospheric effects of the acquired data are essential for precise and reliable vegetation analysis in RS applications [42].

Moreover, it is important to mention that the effects of atmospheric conditions are amplified when the multispectral sensor is deployed on a satellite. This is because light must pass through the atmosphere layers twice. In the case of UAVs, instead, since the flight altitude is low, typically around 100 m, the effect of the part of the atmosphere below the altitude of the UAV is negligible compared to the total effect of the atmosphere; therefore, only the single path from the Sun to the ground is to be considered.

Spectral radiance, denoted in W ${\mathrm{sr}}^{-1}$ $m^{-2}$ ${\mu m}^{-1}$, serves as a measure of the energy flux detected by a sensor in a specific wavelength. For practical storage and analysis, these radiance values are transformed into digital numbers (DNs), i.e., the pixel values. The bit-depth employed for the representation of DNs is dependent on the sensor type, encompassing 6 bits or 7 bits for MultiSpectral Scanner (MSS) sensors, 8 bits for the DJI P4-multispectral camera, Thematic Mapper (TM), and Enhanced Thematic Mapper Plus (ETM+) sensors, and 12 bits for the Landsat 8 sensors, as elucidated by the paper [34].

Reflectance is crucial in RS applications as it facilitates the analysis of various materials and surfaces based on their spectral responses. Researchers often utilize reflectance data to distinguish and interpret features in multispectral or hyperspectral imagery, enabling the extraction of valuable information about the Earth’s surface. Reflectance is a dimensionless metric that denotes the ratio of the radiation reflected by an object measured by the camera sensor, $L_{s}\left(\lambda \right)$, to the incident radiation upon it, $L_{i}\left(\lambda \right)$ [31]:

(1) $$L_{S}\left(\lambda \right)=\rho \left(\lambda \right)\;\cdot \;L_{i}\left(\lambda \right)$$

Here, $L_{i}\left(\lambda \right)$ is evaluated from the solar radiance, $S_{ir}\left(\lambda \right)$, multiplied by the atmospheric effect response, $H_{a}\left(\lambda \right)$:

(2) $$L_{i}\left(\lambda \right)=S_{ir}\left(\lambda \right)\;\cdot \;H_{a}\left(\lambda \right)$$

The reflectance spectrum, denoted as ρ(λ), characterizes the material properties of an object and is independent of the illumination conditions. By analyzing the object’s reflectance spectrum, ρ(λ), it becomes possible to identify materials within a scene. This identification process involves comparing the reflectance spectra of the scene with a pre-existing library of known spectra [31].

### 2.3. Proposed Workflow

**Figure 2:**
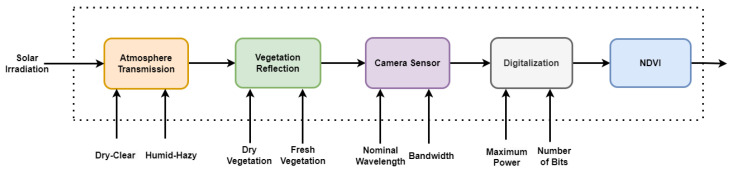
The proposed workflow in this paper for the NDVI evaluation incorporates the modeling of solar irradiation and atmospheric transmission under various conditions (including dry–clear and humid–hazy scenarios), vegetation reflection corresponding to both dry and fresh leaf states, as well as the performance of the camera sensor and digitization step.

This paper introduces a preliminary workflow, illustrated in Figure 2, that integrates essential factors crucial for evaluating the NDVI. The workflow encompasses the modeling of solar irradiation, atmospheric transmission under various conditions, including dry–clear and humid–hazy scenarios, vegetation reflection corresponding to both dry and fresh leaf statuses, and the performance of the camera sensor. As mentioned above, in this workflow, the effect of the atmospheric transmission is considered in the single path between the Sun and the ground, since the effect of the atmospheric transmission on the reflected light is considered negligible. For the camera sensor, the variation in the nominal wavelength, with a specified and unchanging bandwidth of 20 nm, is assumed. The workflow is based on the assessment, which involves the evaluation of the incident OD captured by the camera and its conversion to digital format through 8-bit quantization. During the digitalization stage, the SNR of the camera is applied, and the resultant digital data are subsequently employed for the computation of the NDVI.

In the following sub-sections, each step of the proposed workflow is detailed.

#### 2.3.1. Solar Irradiation

**Figure 3:**
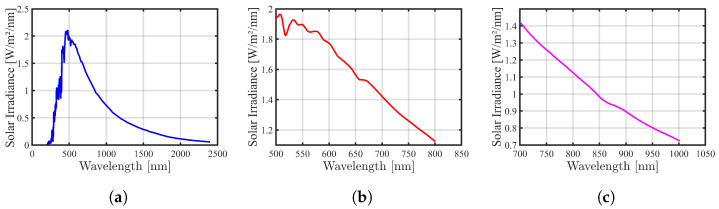
SSI measured by SIM at 1 AU (24 h average) 15 March 2018 00:00 Z to 24:00 Z [43]. (**a**) SSI over the 200 nm–2400 nm; (**b**) SSI around the R band; (**c**) SSI around the NIR band.

To model the Solar Spectral Irradiance (SSI), a publicly available dataset sourced from measurements obtained by the Spectral Irradiance Monitor (SIM) instrument is used [43]. The dataset comprises averaged measurements over a 24 h period, covering a spectral range from 200 nm to 2400 nm, with a spectral resolution ranging from 2 nm (for wavelengths lower than 0.28 μm) to 45 nm (for wavelengths higher than 0.4 μm). These measurements were acquired at a mean solar distance of 1 Astronomical Unit (AU) and zero relative line-of-sight velocity concerning the Sun. The absolute uncertainty of the SIM instrument to measure the SSI is approximately 0.2%. The dataset contains the Julian day, minimum wavelength, maximum wavelength, instrument mode, data version number, irradiance value, irradiance uncertainty, and data quality. Figure 3a illustrates the measured SSI averaged over a period of 24 h.

To fulfill the modeling objectives, spectral bands around the R and NIR wavelengths of the SSI are selected. The best-fitting curves for these spectral bands were determined utilizing the linear interpolation function, implemented through MATLAB software tools utilizing MATLAB R2023b. The curves obtained from this process for R and NIR are depicted in Figure 3b,c.

#### 2.3.2. Atmosphere Transmission

To accurately model the behavior of solar radiation at the ground surface, it is imperative to consider the influence of the atmosphere on solar irradiance and estimate the atmospheric impact for inclusion in the modeling process.

In the wavelength range below 2500 nm, incident solar radiation undergoes various influences. These include (i) absorption by well-mixed gases such as ozone (O_3_), oxygen (O_2_), methane (CH_4_), and carbon dioxide (CO_2_); (ii) absorption by water vapor; (iii) scattering by molecules; and (iv) scattering and absorption by aerosols and hydrometeors [18]. Gas absorption can substantially alter the received solar flux, with effects being prominent at different wavelengths. In contrast, aerosol absorption, considered a smoothly varying continuous function, typically results in limited absorption loss, varying from maritime aerosols to urban aerosols rich in carbon. Minimum absorption is observed for maritime aerosols, and maximum absorption is prevalent in urban aerosols containing a significant carbon content [18]. Scattering, whether from molecules or aerosols, contributes to the modification of the incident solar flux. Molecular scattering effects are significant in the visible/near-infrared (VNIR) range, diminishing at longer wavelengths. Aerosol scattering has a noticeable impact on the VNIR/short-wave infrared (SWIR) range, particularly due to larger aerosol particle sizes [18].

**Figure 4:**
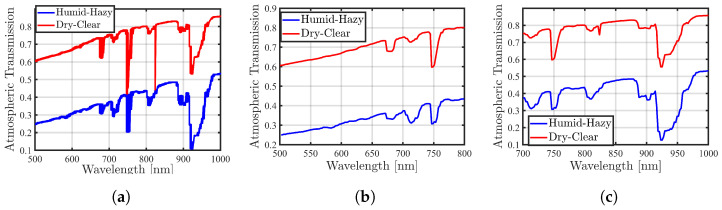
(**a**) The atmospheric transmission in the spectral range of 500 nm–1000 nm is plotted for two conditions (dry–clear in red and humid–hazy in blue colors). This plot results from contributions to the overall transmission by mixed gases, aerosols, and water vapor, with data estimated from the paper [18]. Accordingly, (**b**) depicts the spectral wavelength around the R band, while (**c**) shows the spectral wavelength around the NIR band.

Figure 4a, created from the graph mentioned in [18], illustrates atmospheric transmittance for several significant gaseous, water vapor, and aerosol absorption features observed in the spectral region of 400 nm–2500 nm under two atmospheric conditions: dry–clear and humid–hazy. For modeling atmospheric transmission, the spectral range of 500 nm–1000 nm is selected and shown in Figure 4a. In this spectral range, ozone exhibits weak absorption in the interval of 500 nm–700 nm, while a strong and narrow oxygen absorption line is present at 760 nm. Water vapor absorption in the VNIR spectrum is characterized by numerous bands of varying strengths and spectral widths. Two very weak absorption bands are present at 600 nm and 660 nm, while slightly stronger absorption and more significant bands are located at 730 nm, 820 nm, and 910 nm. Additionally, a distinct and robust water vapor absorption is observed at 940 nm. To achieve the modeling goals, spectral bands around the R and NIR wavelengths of atmospheric transmission were chosen. Utilizing the linear interpolation function with MATLAB software tools, the fitting curves for these selected spectral bands were determined. The resulting curves for R and NIR can be observed in Figure 4b,c.

#### 2.3.3. Modeling Transmission of Solar Irradiance through the Atmospheric Conditions

**Figure 5:**
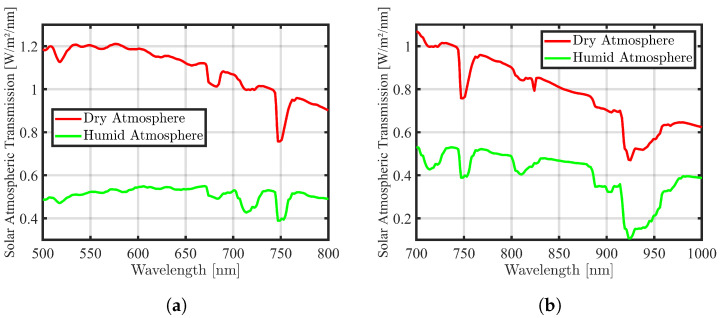
Solar irradiation is affected by two atmospheric conditions (dry–clear in red and humid–hazy in green colors), (**a**) in the R band and (**b**) in the NIR band.

The mathematical curve models derived from Section 2.3.1 and Section 2.3.2 provide essential representations of solar irradiance and atmospheric transmission, respectively. By combining these models, the study gains a comprehensive understanding of how the atmosphere influences the transmission of solar irradiance, considering variations in dry–clear and humid–hazy conditions. To incorporate these impacts, the mathematical curve models are multiplied together, separately for the R and NIR bands in dry–clear and humid–hazy air conditions [44]. The curves are sampled at specific wavelengths with a resolution of 1 nm. Subsequently, the corresponding data points of the two curves are multiplied numerically in an elementwise manner. The results of these calculations are depicted in Figure 5, which offers insights into the dynamics of solar radiation as it travels through the atmospheric layers of the Earth.

#### 2.3.4. Vegetation Reflection

**Figure 6:**
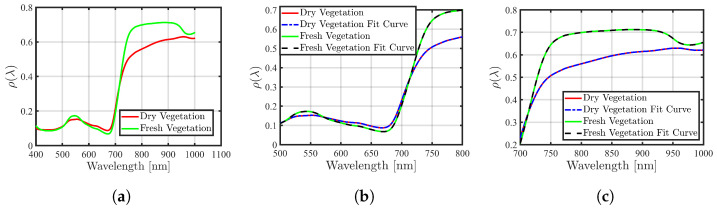
Spectral reflectance of dry and fresh vegetation. (**a**) In the wavelength range of 400 nm–980 nm, (**b**) in the R band with its fit curve, and (**c**) in the NIR band with its fitted curve.

To model vegetation reflection, the Accelerated Canopy Chemistry Program (ACCP) dataset containing VNIR reflectance spectra for both fresh and dry Douglas fir leaves is utilized [45]. This dataset covers the spectral range of 400 nm–2498 nm at 2 nm intervals and a resolution of 10 nm. Selected reflectance spectra of a sample leaf in both dry and fresh states in the range of 400 nm–980 nm are displayed in Figure 6a. The reflection data for the R and NIR bands were extracted. To formulate a mathematical model of leaf reflection for both dry and fresh states, fitting curves were derived through linear interpolation using *MATLAB* tools. The resulting curves are evaluated and plotted alongside the corresponding data in Figure 6b,c.

#### 2.3.5. Vegetation Reflection Affected by Solar Irradiance Passing through the Atmosphere Condition

**Figure 7:**
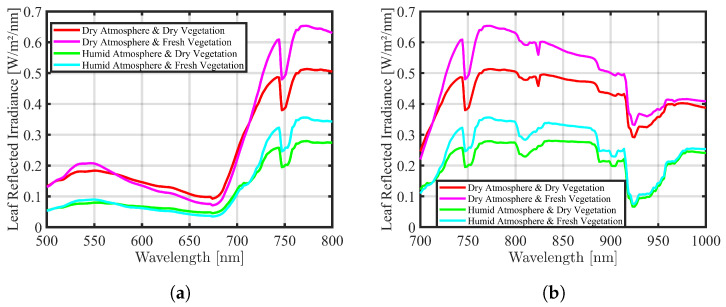
The spectral reflected irradiance of dry and fresh vegetation is affected by two atmospheric conditions (dry–clear and humid–hazy) in both (**a**) the R band and (**b**) the NIR band.

To derive the reflection of vegetation considering incident light from solar irradiance passing through the atmosphere under dry–clear and humid–hazy air conditions, it is necessary to multiply the mathematical curves obtained from Section 2.3.3 (representing solar irradiance affected by atmospheric conditions) with the leaf reflection curve derived from Section 2.3.4. By multiplying these mathematical curves, an estimation of the impact of solar irradiance and atmospheric conditions on vegetation reflection is evaluated [44]. The curves were sampled at specific wavelengths with a resolution of 1 nm. Then, the corresponding data points of the two curves were multiplied elementwise numerically. The results of this estimation are depicted in Figure 7 for dry and fresh leaves under both dry–clear and humid–hazy atmospheric conditions in the R and NIR bands separately.

#### 2.3.6. Camera Sensor

In the experimental scenario, a DJI P4-multispectral camera, equipped with six 1/2.9 CMOS sensors [46], was assumed to be used for the acquisition of experimental data for vegetation-reflected irradiance affected by the atmospheric conditions. The camera comprises an RGB sensor for visible light images and five monochrome sensors dedicated to multispectral image acquisition. The filters used in the camera have specific wavelength ranges, with R: 650 nm ± 16 nm, and NIR: 840 nm ± 26 nm. The bandwidth of a multispectral camera ranges from 15 nm to 30 nm [47]; thus, in the analysis performed, it was assumed to be 20 nm for both the R and the NIR bands. Notably, the DJI P4-multispectral camera is configured with non-overlapping filter bands for the R and the NIR bands, ensuring distinct spectral information capture for accurate multispectral analysis.

#### 2.3.7. Digitalization

The DJI P4-multispectral camera functions by capturing energy from the real scene and converting it into digital numbers for each pixel. The bit-depth of the DJI P4-multispectral camera is 8, with a numerical range between 0 and 255. In order to model the digitalization function, knowledge about the maximum energy in the real scenario is crucial. To evaluate the maximum energy for the R and NIR bands separately, an empty scene on the Earth’s surface is considered, where solar irradiance passes through the atmospheric conditions to reach the ground surface, completely reflected at the vacant ground surface with no presence of vegetation.

The maximum energy for the R band is obtained by considering the reflected irradiance in the wavelength range of 614 nm to 686 nm within a sensor bandwidth of 20 nm. The energy under the curve, multiplied by solar irradiance as evaluated in Section 2.3.1, and atmospheric conditions (dry–clear or humid–hazy, separately) as assessed in Section 2.3.2, are then computed. The maximum energy density obtained for the R band is 22.962 W $m^{-2}$.

A similar procedure was performed to determine the maximum energy for the NIR considering a wavelength range from 794 nm to 886 nm with a bandwidth of 20 nm. The resulting outcome yields the maximum energy for the NIR, computed to be 16.908 W $m^{-2}$.

#### 2.3.8. NDVI

Green vegetation contains chlorophyll, a chemical compound that selectively absorbs radiation in the R and blue wavelengths and reflects radiation in the green and NIR wavelengths. Monitoring reflectance in the R region of the electromagnetic spectrum (620 nm–750 nm) facilitates the assessment of solar radiation absorption by chlorophyll. Additionally, analyzing reflectance in the NIR range (750 nm–1300 nm) provides information about the maximum energy reflected by leaf cell structures, offering insights into the health status of vegetation. The NDVI serves as a widely utilized vegetation metric for monitoring the health state of vegetation and can be calculated as the following equation:

(3) NDVI=(NIR−R)/(NIR+R),

where NIR and R represent the quantized optical density of solar irradiance reflected by the object in the NIR and R spectral bands.

The NDVI values range from −1 to +1, with values below 0.2 indicating the absence of vegetation, typically representing exposed soil, rocks, sands, or concrete surfaces. Negative values are associated with water or urban areas. Values near 1 suggest denser and healthier vegetation, encompassing crops, shrubs, grasses, and forests [31,48,49].

### 2.4. Sensitivity Assessment

Sensitivity analysis assesses the influence of various values of independent variables on a specific dependent variable within a defined set of assumptions. This study involves comprehending how the variation of atmospheric conditions, as well as instrumental parameters, by altering the design parameters or operational conditions, affects multispectral images in the measurement of the NDVI. In this regard, Monte Carlo analyses are employed to evaluate uncertainties arising from the atmospheric conditions and camera sensor factors and identify how each of these factors contributes individually to the overall uncertainty in measuring the NDVI.

#### Monte Carlo Simulation

**Figure 8:**
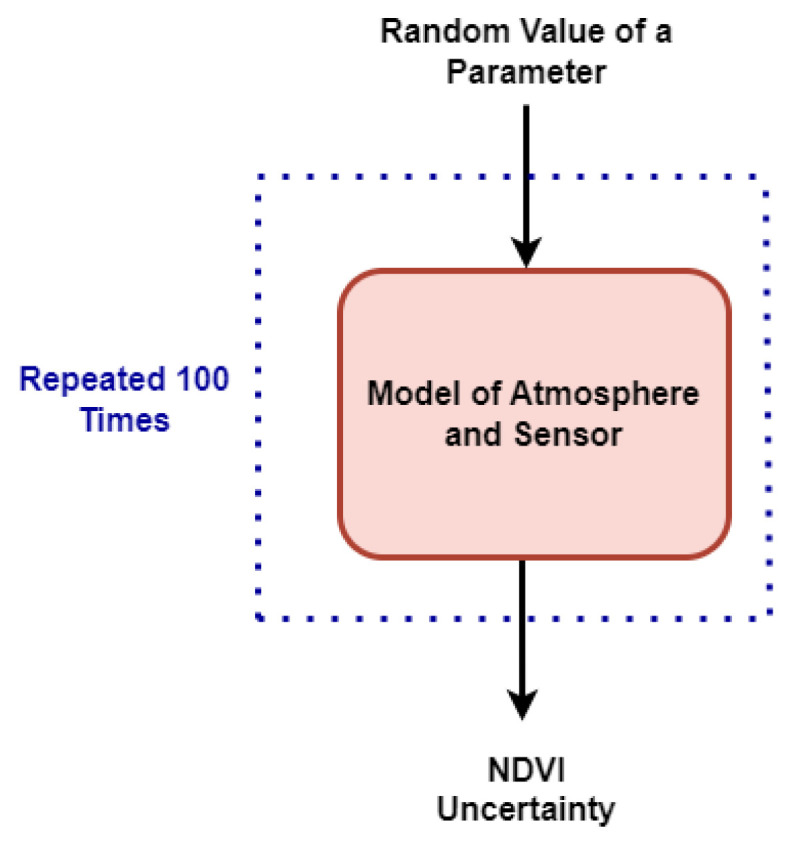
Sensitivity assessment of the workflow proposed in this paper through Monte Carlo analysis.

The methodology for uncertainty evaluation in this research follows the principles outlined in the Guide to the Expression of Uncertainty in Measurement (GUM) [50], specifically adopting the Monte Carlo simulation approach. Monte Carlo simulation is employed to estimate uncertainty and obtain the distribution of data for each pixel value in multispectral images according to the workflow depicted in Figure 2. As depicted in Figure 8, uncertainty evaluation is conducted through Monte Carlo simulations with 100 observations against random variations of each influence factor and by considering a combination of them, from solar irradiance to the camera sensor characteristics.

## 3. Results

The uncertainty evaluation of the NDVI measurement is related to understanding the impact of parameter variations at each stage of the workflow, as shown in Figure 2. To achieve this understanding, the experiment was conducted in six parts, namely (i) NDVI uncertainty vs the nominal wavelength, (ii) NDVI uncertainty vs the solar irradiance standard deviation, (iii) NDVI uncertainty vs the camera SNR, (iv) NDVI uncertainty vs the leaf state, (v) NDVI uncertainty vs all uncertainty sources, and (vi) a comparison with another camera sensor. These assessments were conducted on solar irradiance (Section 2.3.1) under various air conditions described in Section 2.3.2, utilizing datasets comprising both dry and fresh leaves (Section 2.3.4), and employing a camera sensor (Section 2.3.6). Detailed explanations of each part and their respective results are provided in the following subsections.

### 3.1. NDVI Uncertainty vs. Nominal Wavelength

In the experiment, one dried and one fresh leaf were selected. For Monte Carlo analysis, a loop with 100 trials was employed, wherein the nominal wavelength was randomly changed based on the nominal wavelength tolerance of the camera. This random value was selected from a uniform distribution function. Then, the energy of the multiplied curves, encompassing (i) solar irradiance without considering its standard deviation, (ii) atmospheric transmission, and (iii) leaf reflection in both the R and NIR spectra, as mentioned in Section 2.3.5, was assessed. After evaluating the energy, a quantization function for 8 bits, considering the maximum energy evaluated separately in the R and NIR, as mentioned in Section 2.3.7, was applied. Subsequently, an unsigned integer value was computed for each wavelength in the R and NIR. The NDVI value was calculated using these two integers, based on the formula explained in Section 2.3.8. Out of the 100 trials, 100 NDVI values were used to evaluate the effect of the uncertainty source on the NDVI values.

**Figure 9:**
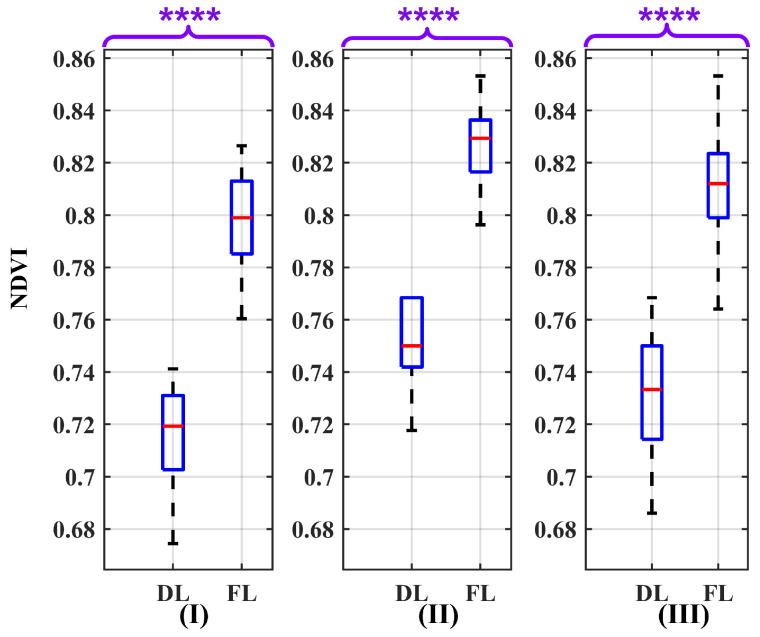
Box plots illustrating the effect of nominal wavelength as a source of uncertainty on the NDVI values for the atmospheric conditions: (**I**) dry–clear, (**II**) humid–hazy, and (**III**) mixed, considering both dry leaves (DLs) and fresh leaves (FLs). The *p*-value results of the Wilcoxon Rank Sum Test are significantly below 0.0001 (*p* ≤ 0.0001). Therefore, to report them, four asterisks are used to denote the level of significance. The red line in each box represents the median (Med) of NDVI values in related conditions.

To precisely investigate the degree of this effect, it is imperative to determine the distribution of the NDVI values. The chi-squared test was applied to the NDVI values, revealing that the NDVI does not follow a Gaussian distribution. Moreover, the NDVI values of dry and fresh leaves are independent. As the NDVI values are independent and do not conform to a Gaussian distribution, the Wilcoxon Rank Sum Test, also recognized as the Mann–Whitney U test, was employed. This non-parametric test, which does not assume known distributions and avoids dealing with parameters, is utilized to assess the extent of overlap of the NDVI values, determining whether the NDVI values for distinct leaves are likely to originate from the same population. In this test, the null hypothesis (H0) is that two input data are samples from the same distribution with identical medians, while the alternative hypothesis (H1) assumes they are not. Rejecting the H0 implies evidence of a shift in one distribution relative to the other and provides evidence that the medians of the two populations differ. Moreover, the *p*-value indicates the probability of observing the data if the H0 is true. The outcome of this test is depicted in Figure 9 for each separate air condition. The resultant *p*-values were significantly below 0.0001 (*p* ≤ 0.0001), so to report them, four asterisks are used to denote the level of significance. This level of significance implies strong evidence to reject the H0 in favor of the H1. Therefore, there is a difference between dry and fresh leaves under different conditions. With a more reliable and trustworthy multispectral camera and data about the leaf chemical composition and species, it is possible to distinguish the leaves’ status.

Table 1 presents the conclusive outcomes of the conducted evaluation, incorporating the median (Med) NDVI along with its associated first quartile (Q1), third quartile (Q3), and Interquartile Range (IQR) for leaf state (LS) in both dry and fresh leaves under various atmospheric conditions (Atm. Cond.). These conditions include humid–hazy, dry–clear, and a mix of air conditions. The variability of the NDVI with respect to fresh leaves, assessed through the IQR, was determined to be 0.028, while for dry leaves, it was measured as 0.036. Despite considering the nominal wavelength as a potential source of uncertainty, these values exhibit low magnitudes, indicative of the stability of the NDVI values. Also, the table includes the distance between the Q1 of the fresh leaves ($Q1_{F}$) and the Q3 of the dry leaves ($Q3_{D}$) in the same air condition, providing information about the ability to distinguish between the two leaf conditions. In the context of distinguishing between dry and fresh leaves, the Med of fresh leaves consistently surpassed that of dry leaves. Consequently, the assessment of the distance of the leaf condition involved quantifying between $Q1_{F}$ and $Q3_{D}$. A greater distance indicates a higher ability to distinguish the leaves under different conditions based on the NDVI values and setting a threshold. By setting a threshold of the NDVI value around 0.775, it is possible to distinguish between dry and fresh leaves under various air conditions. However, in a dry–clear atmosphere, the threshold can be below 0.775, while in a humid–hazy atmosphere, it can be above 0.775. Therefore, the choice of the threshold depends on the atmospheric conditions. Moreover, the distance, $Q1_{F}-Q3_{D}$, in different air conditions shows the highest value in dry–clear atmospheric conditions, which is 0.054, compared to in humid–hazy and mixed air conditions, which is 0.049. Therefore, the ability to differentiate between leaf states in a dry atmosphere is more feasible.

### 3.2. NDVI Uncertainty vs. Solar Irradiance Standard Deviation

**Figure 10:**
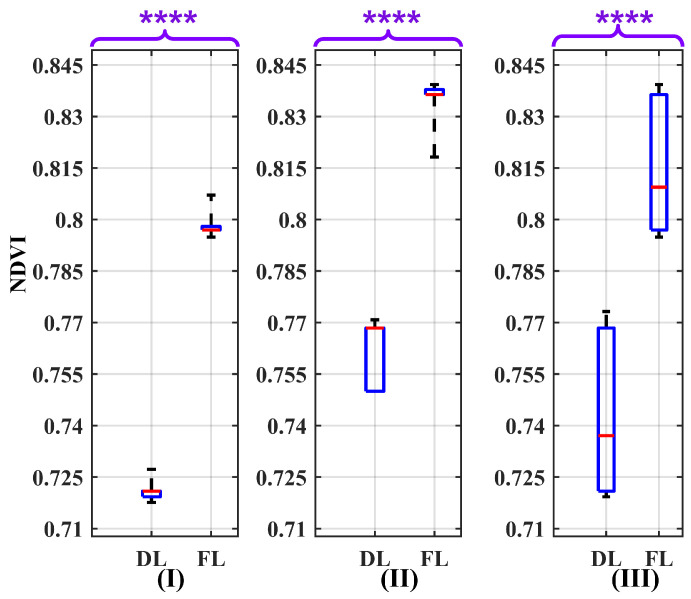
Box plots illustrating the effect of the variability in solar irradiance measurement as a source of uncertainty on the NDVI values for the atmospheric conditions: (**I**) dry–clear, (**II**) humid–hazy, and (**III**) mixed, considering both dry leaves (DLs) and fresh leaves (FLs). The *p*-value results of the Wilcoxon Rank Sum Test are significantly below 0.0001 (*p* ≤ 0.0001). Therefore, to report them, four asterisks are used to denote the level of significance. The red line in each box represents the median (Med) of NDVI values in related conditions.

For this test, the same procedure as mentioned in Section 3.1 was repeated. However, the difference lies in the initial step, where data regarding the uncertainty of the solar irradiance instrument for each wavelength within the dataset mentioned in [43] were added, along with white Gaussian noise, to the solar irradiance curve. Furthermore, the nominal wavelength was fixed to match the camera’s nominal wavelength in the R and NIR spectra. The outcome of Wilcoxon Rank Sum Test on the NDVI values is presented in the box plot shown in Figure 10. The obtained *p*-value is markedly below 0.0001 (*p* ≤ 0.0001), which signifies robust evidence for rejecting the H0 in favor of the H1. Furthermore, the absence of any overlap between the boxes in Figure 10 indicates the feasibility of distinguishing between dry and fresh leaves under various conditions.

The conclusive outcomes of the conducted assessment are presented in Table 2. The IQR values indicate the NDVI variability for fresh and dry leaves as 0.039 and 0.047, respectively. Despite accounting for the standard deviation of solar irradiance as a potential source of uncertainty, these variation values exhibit low magnitudes, signifying the stability of the NDVI values.

Moreover, the table incorporates the $Q1_{F}$ to $Q3_{D}$ distance in the same air condition, providing insights into the capability to differentiate between the two leaf conditions. By establishing a threshold for the NDVI value within the range of 0.777 to 0.787, it becomes feasible to discern between dry and fresh leaves under various air conditions. In a dry–clear atmosphere, the threshold can shift slightly downward, and in a humid–hazy atmosphere, it can shift slightly upward. Furthermore, the $Q1_{F}-Q3_{D}$ distance across different air conditions reveals a higher magnitude in dry–clear atmospheric conditions (0.076) compared to humid–hazy (0.068) and mixed air conditions (0.029). As a result, the practicality of distinguishing between leaf states in a dry atmosphere is enhanced.

### 3.3. NDVI Uncertainty vs. Camera SNR

The signal in a pixel is quantified by the total number of detected photoelectrons, and a higher quantum efficiency or larger pixel size yields an increased signal due to a higher count of photoelectrons [51]. The SNR measures the relationship between the useful signal and unwanted background noise within a pixel. In various embedded vision applications (crop detection), particularly those involving edge-based processing with artificial intelligence or ML algorithms analyzing processed images for intelligent decision making, a high SNR is essential to deliver detailed and accurate image outputs [52]. The SNR varies depending on the device, and based on literature reviews, an SNR range from 40 dB to 60 dB has been measured for multispectral cameras [52,53]. This range is estimated to be suitable for maintaining a balance between signal strength and noise levels.

In this test, the same procedure as mentioned in Section 3.1 was repeated, using a fixed camera’s nominal wavelength in the R and NIR. During the quantization function step, the SNR value, along with an additive white Gaussian noise, is incorporated into the quantization function, as mentioned in Section 2.3.7, for the conversion of analog data to digital. The SNR value was initialized at 40 dB and incremented by 10 dB in each iteration of the Monte Carlo analysis until reaching 60 dB.

**Figure 11:**
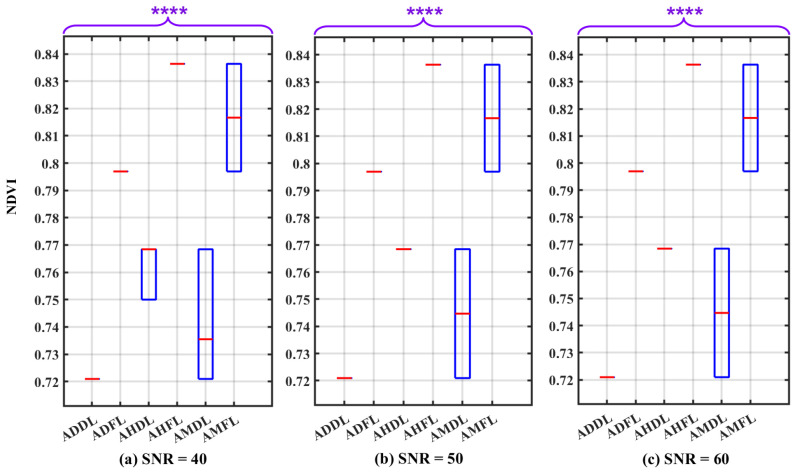
Box plots illustrating the influence of the camera sensor under three different SNR conditions: (**a**) SNR = 40 dB, (**b**) SNR = 50 dB, and (**c**) SNR = 60 dB, serving as uncertainty factors on the NDVI values. The plot considers various atmospheric conditions and leaf states, including: atmosphere dry–clear and dry leaf (ADDL), atmosphere dry–clear and fresh leaf (ADFL), atmosphere humid–hazy and dry leaf (AHDL), atmosphere humid–hazy and fresh leaf (AHFL), atmosphere mixed for dry leaf (AMDL), and atmosphere mixed for fresh leaf (AMFL). The *p*-value results of the Wilcoxon Rank Sum Test are significantly below 0.0001 (*p* ≤ 0.0001). Therefore, to report them, four asterisks are used to denote the level of significance. The red line in each box represents the median (Med) of NDVI values in related conditions.

The results of the Wilcoxon Rank Sum Test on the NDVI values are depicted in the box plots shown in Figure 11 for SNR values of 40 dB, 50 dB, and 60 dB, respectively. These box plots clearly indicate the absence of any overlap between the boxes. The computed *p*-value, significantly below 0.0001, provides robust evidence for rejecting the H0 in favor of the H1. This outcome thereby enables the differentiation of leaf states under varying air conditions.

Table 3 presents the definitive outcomes of the conducted assessment for SNR values of 40 dB, 50 dB, and 60 dB. The IQR for NDVI variability in fresh leaves is 0.039, and for dry leaves, it is 0.047. The analysis indicates that different SNR values do not influence significantly the degree of NDVI variation. Considering camera SNR as a potential source of uncertainty, the variation of NDVI values displays low magnitudes, indicative of the stability of NDVI values.

By establishing a threshold of the NDVI value between 0.770 and 0.790, it becomes feasible to distinguish between dry and fresh leaves under various air conditions. In dry conditions, the threshold can shift slightly down; in humid–hazy conditions, it can shift slightly up. In addition, the distance, $Q1_{F}-Q3_{D}$, in different air conditions reveals a higher magnitude in dry–clear atmospheric conditions (0.076) compared to humid–hazy (0.068) and mixed air conditions (0.029). Importantly, varying SNR values do not impact the magnitude of this distance. Also, the practicality of distinguishing between leaf states in a dry atmosphere remains high.

### 3.4. NDVI Uncertainty vs. Leaf State

**Figure 12:**
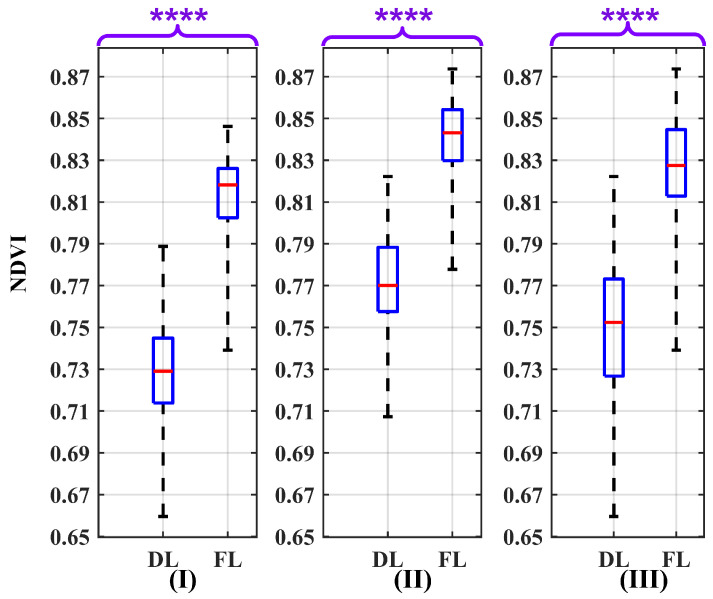
Box plots illustrating the effect of the variability of the NDVI values as a source of uncertainty on the NDVI values for atmospheric conditions: (**I**) dry–clear, (**II**) humid–hazy, and (**III**) mixed, considering both dry leaves (DLs) and fresh leaves (FLs). The *p*-value results of the Wilcoxon Rank Sum Test are significantly below 0.0001 (*p* ≤ 0.0001). Therefore, to report them, four asterisks are used to denote the level of significance. The red line in each box represents the median (Med) of NDVI values in related conditions.

In this sensitivity analysis, the uncertainty due to the binary model of the leaf state (fresh or dry) is assessed. Therefore, for the Monte Carlo analysis, a set of 49 leaf samples was selected for each leaf state. The sample size must meet a minimum requirement of 30, as certain statistical tests, such as the T-squared test, are not applicable for sample sizes below this number. Consequently, 49 leaf samples were chosen, as this quantity is readily available within the dataset [45]. By maintaining a fixed nominal wavelength equivalent to the camera’s nominal wavelength in the R and NIR spectra, the identical procedure outlined in Section 3.1 was utilized. The result of the Wilcoxon Rank Sum Test was employed to elucidate the impact of this uncertainty source on the NDVI values in detail, as depicted in the box plots presented in Figure 12. The derived *p*-value, notably below 0.0001, provides robust evidence for rejecting the H0 in favor of the H1. Moreover, the absence of any overlap between the boxes, in Figure 12, emphasizes the feasibility of discriminating between dry and fresh leaves under varying air conditions.

Table 4 summarizes the conclusive results of the conducted assessment. The IQR values for the NDVI variability in fresh leaves and dry leaves are 0.032 and 0.046, respectively. These values, considering the distribution of measured leaf values in dry and fresh states with respect to the NDVI measurement as a potential source of uncertainty, demonstrate low magnitudes, suggesting the low variability of the NDVI values.

In the context of distinguishing between dry and fresh leaves, setting a threshold around 0.790 for the NDVI value makes it possible to differentiate between them under various air conditions. However, in a dry atmosphere, this threshold can move a bit downward, and in a humid atmosphere, it can move a bit upward. Therefore, selecting the threshold also depends on the atmospheric conditions. Moreover, the $Q1_{F}-Q3_{D}$ distance in different air conditions reveals a higher magnitude in dry–clear atmospheric conditions (0.057) compared to humid–hazy (0.042) and mixed air conditions (0.040). Hence, the feasibility of differentiating between leaf states in a dry atmosphere is increased.

### 3.5. NDVI Uncertainty vs. All Uncertainty Sources

In this section, all the uncertainty sources, including variations in the solar irradiance standard deviation, the camera SNR, the camera nominal wavelength, and the variability of the measurand within each considered leaf state, are applied to the dataset for the assessment of NDVI uncertainty. In the Monte Carlo analysis, a dataset of 49 leaf samples was selected. The solar irradiance standard uncertainty, modeled by a Gaussian distribution function, and the SIM instrument’s uncertainty were applied to the solar irradiance curve before its utilization in the energy evaluation. Variations in the nominal wavelength, introduced through a uniform distribution function, were applied.

**Figure 13:**
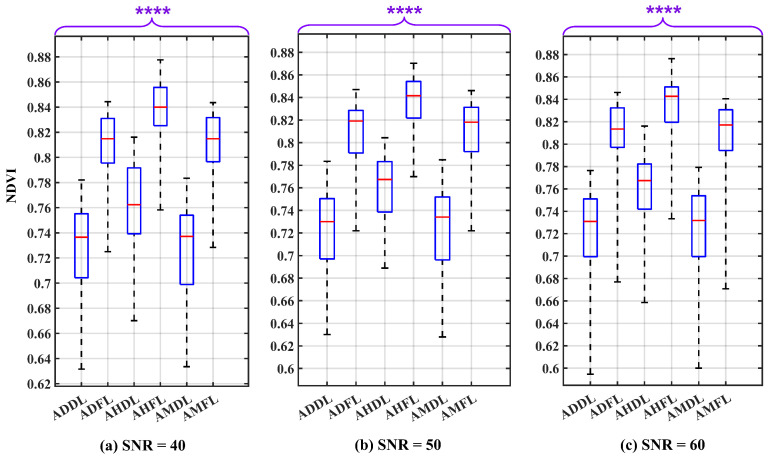
Box plots illustrating the effect of all uncertainty sources under three different SNR conditions: (**a**) SNR = 40 dB, (**b**) SNR = 50 dB, and (**c**) SNR = 60 dB, serving as uncertainty factors on the NDVI values. The plot considers various atmospheric conditions and leaf states, including: atmosphere dry–clear and dry leaf (ADDL), atmosphere dry–clear and fresh leaf (ADFL), atmosphere humid–hazy and dry leaf (AHDL), atmosphere humid–hazy and fresh leaf (AHFL), atmosphere mixed for dry leaf (AMDL), and atmosphere mixed for fresh leaf (AMFL). The *p*-value results of the Wilcoxon Rank Sum Test are significantly below 0.0001 (*p* ≤ 0.0001). Therefore, to report them, four asterisks are used to denote the level of significance. The red line in each box represents the median (Med) of NDVI values in related conditions.

The SNR value was initialized and incremented using the same procedure as mentioned in Section 3.3. Following this, the resulting energy was calculated using the same procedure as that used in Section 3.1. The Wilcoxon Rank Sum Test results to elucidate the extent of the effect of these uncertainty sources on the NDVI values in detail are portrayed in the box plots presented in Figure 13 for SNR values of 40 dB, 50 dB, and 60 dB, respectively. These box plots clearly indicate the absence of overlap between the boxes. The computed *p*-value, significantly below 0.0001, provides robust evidence for rejecting the H0 in favor of the H1. This outcome thereby enables the differentiation of the leaf states under varying air conditions.

Table 5 summarizes the conclusive results of the evaluation with SNR values of 40 dB, 50 dB, and 60 dB. The IQR for the NDVI variability in fresh leaves is 0.043, and for dry leaves, it is 0.053. Different SNR values have a minor impact on the variability. These NDVI variability values show low magnitudes and suggest the stability of the NDVI values.

Additionally, by setting a threshold for the NDVI value just around 0.790 makes it challenging, but possible to distinguish between dry and fresh leaves under various air conditions. In a dry–clear atmosphere, the threshold can shift down a bit, while in a humid–hazy atmosphere, it can shift up a bit. Furthermore, the distance, $Q1_{F}-Q3_{D}$, in various air conditions, reveals a higher magnitude in dry–clear atmospheric conditions (0.047) than in humid–hazy and mixed air conditions, being 0.040, and different SNR values have a negligible effect on this distance. Therefore, the feasibility of differentiating between leaf states in a dry atmosphere is higher.

### 3.6. Comparison with Another Camera Sensor

**Figure 14:**
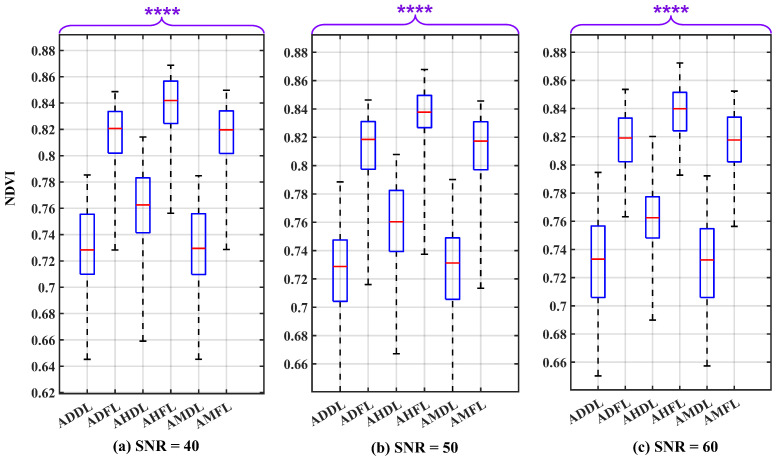
Box plots illustrating the effect of all uncertainty sources by using the AQ600 camera features under three different SNR conditions: (**a**) SNR = 40 dB, (**b**) SNR = 50 dB, and (**c**) SNR = 60 dB, serving as uncertainty factors on the NDVI values. The plot considers various atmospheric conditions and leaf states, including: atmosphere dry–clear and dry leaf (ADDL), atmosphere dry–clear and fresh leaf (ADFL), atmosphere humid–hazy and dry leaf (AHDL), atmosphere humid–hazy and fresh leaf (AHFL), atmosphere mixed for dry leaf (AMDL), and atmosphere mixed for fresh leaf (AMFL). The *p*-value results of the Wilcoxon Rank Sum Test are significantly below 0.0001 (*p* ≤ 0.0001). Therefore, to report them, four asterisks are used to denote the level of significance. The red line in each box represents the median (Med) of NDVI values in related conditions.

The proposed workflow has been evaluated considering another multispectral sensor available on the market, the AQ600 multispectral camera [54], composed of five spectral channels of 3.2M pixels each, along with one 12.3M pixel RGB channel. It features a sapphire optical window, a large aperture, low distortion, broad-spectrum transmission, and an all-glass lens. The AQ600 offers advantages such as fast imaging speed, high resolution, and accurate image quality, making it more suitable for VTOL and fixed-wing UAVs. The filters used in the camera have specific wavelength ranges, R: 660 nm ± 22 nm and NIR: 840 nm ± 30 nm. Its resolution is 12 bits for the multispectral camera and 8 bits for the RGB channels. In the evaluation, the integration of all uncertainty sources, following the procedure outlined in Section 3.5 with 12-bit quantization, was applied. Subsequently, the Wilcoxon Rank Sum Test was conducted to thoroughly examine the NDVI values, with a detailed portrayal in the box plots presented in Figure 14 for SNR values of 40 dB, 50 dB, and 60 dB, respectively. The computed *p*-value, significantly below 0.0001, provides robust evidence for rejecting the H0 in favor of the H1. This outcome facilitates the differentiation of the leaf states under varying air conditions. Table 6 presents the comprehensive findings of the evaluation across SNR values of 40 dB, 50 dB, and 60 dB. The IQR for NDVI variability in fresh leaves is 0.038, and for dry leaves, it is 0.059. Minor fluctuations in NDVI variability are observed with varying SNR values, indicating the stability of the NDVI measurements. Moreover, the difference between $Q1_{F}$ and $Q3_{D}$ within the same atmospheric condition is slightly higher in dry–clear atmospheric conditions (0.050) compared to humid–hazy and mixed air conditions, which measure 0.047 and 0.048. Different SNR values have a negligible impact on this difference. Establishing a threshold for the NDVI value around 0.790 enables the distinction between dry and fresh leaves under diverse air conditions. In dry–clear atmospheres, the threshold may shift downward slightly, while in humid–hazy atmospheres, it may shift upward marginally. In comparing the DJI P4-multispectral and AQ600 cameras, despite differences in their quantization level, number of bits, and filter bands in the R and NIR, it is feasible to differentiate between leaf states, and the NDVI values exhibit changes on the order of a few thousandths, indicating that the cameras’ features contribute less to the overall uncertainty compared to other sources.

## 4. Discussion

Looking at the box plot reported in Figure 9, Figure 10, Figure 11, Figure 12, Figure 13 and Figure 14, it is possible to observe that, when only one of the uncertainty sources is considered, the median values referring to the same leaf state and in the same atmospheric conditions are distributed around similar values. On the contrary, the median values referring to the same leaf state show a significant change when the atmospheric condition changes. This demonstrates that the atmospheric conditions introduce a bias to the NDVI measurements.

In PA applications, it is often important to classify the state of the crop. In this paper, for each considered uncertainty source, after having determined the distribution of the NDVI values, the effect on a possible classification was evaluated. This is helpful in anticipating the accuracy of a classifier working on the measurements affected by uncertainty, according to the proposed workflow.

The classification must consider the bias introduced by the atmospheric conditions, provided that they are stable throughout the survey. Therefore, the threshold for the classification should be defined depending on the atmospheric conditions.

The bias introduced by the atmospheric effects could be corrected either by identifying the model of the atmospheric transmission or using a radiometric compensation [55]. This correction is needed when the analysis is carried out on data acquired in different weather and light conditions, such as in the case of multi-temporal comparison [56], or when parts of the field are occluded by clouds or shadows [57,58]. The atmospheric correction allows minimizing the bias and, therefore, the median values of the samples even when they are obtained in different environmental conditions. This will allow a classifier threshold to be set independent of the atmospheric conditions.

Several methods can be used to compensate for atmospheric effects. The Dark Object Subtraction (DOS) method identifies regions within an image with minimal reflectance, typically shadowed areas [58] or water bodies, and subtracts their radiance values to diminish cloud and atmospheric contributions. Advanced radiative transfer models, exemplified by MODTRAN [59], facilitate precise correction by simulating the complex interactions of electromagnetic radiation with atmospheric components. Additionally, reference targets strategically positioned in the scene such as Lambertian sheets [28] and empirical line compensation methodologies establish correlations between sensor readings and ground-truth reflectance values, enabling the accurate adjustment of sensor data [60,61]. Spectral matching techniques further refine calibration efforts by ensuring consistency across different sensors [61].

Even though using radiometric compensation may mitigate the variation of solar irradiance, it has some limitations. Firstly, some radiometric compensation methods are not cost effective for UAV applications, such as the physical-based methods [17], which require the use of several sensors to estimate the atmospheric conditions. Secondly, the empirical linear methods assume a linear behavior of the multispectral camera sensors, which is not always valid [60], and usually, the compensation is performed once during a flight mission with reference targets; thus, they are not able to follow possible changes of the atmospheric conditions [60].

## 5. Conclusions

Utilizing advanced modern information technology in PA enables precise measurement of vegetation, soil, and environmental parameters. This study introduces a workflow for evaluating the effect of uncertainty sources in NDVI measurements, taking into account the OD captured by a multispectral camera within its variation of the nominal working wavelength and limited bandwidth, as well as the camera SNR, solar irradiance standard uncertainty, atmospheric conditions, and considering the variability of the NDVI within the two considered leaf states. By utilizing Monte Carlo simulations, the effect of each uncertainty source and their combined impact on the NDVI measurement was evaluated. The Wilcoxon Rank Sum Test was used to examine the overlap of the NDVI values between dry and fresh leaves under diverse air conditions after uncertainty sources were applied. The results indicate an absence of overlap, so, by establishing a threshold, it is possible to distinguish between fresh and dry leaves under different air conditions. However, this threshold is changed when the environmental conditions change, and it is dependent on the atmospheric conditions.

Furthermore, the results of the experimental section showed that, although uncertainty sources affect the NDVI measurement, the variation of the NDVI values (based on the IQR values) is not significant; therefore, the NDVI values remained stable. In addition, the results highlighted that the variability of the NDVI within the two considered leaf states, atmospheric conditions, and the camera sensor’s nominal wavelength as sources of uncertainty significantly affected the NDVI measurements. On the other hand, the other uncertainty sources, the solar standard deviation and camera SNR, had minimal effects on the NDVI measurements. This comprehensive assessment provides valuable insights suggesting that experts should collect data in dry atmospheric conditions rather than humid ones, particularly when radiometric and atmospheric compensation methods are not employed. Furthermore, it is recommended not to invest additional funds in acquiring a multispectral camera with superior radiometric resolution accuracy compared to the DJI P4-multispectral camera utilized in this study, as the results do not significantly differ, as shown in Section 3.6. It is important to mention that neither radiometric compensation nor any other form of compensation for atmospheric effects were used in this study.

For future studies, it is advised to explore modeling the effects of different uncertainties like the flight conditions (altitude and image overlap), environmental factors (temperature and humidity), sensor tilt, and other relevant factors. These aspects can significantly affect the accuracy of NDVI measurements. Moreover, it would be valuable to assess how different compensation approaches affect these uncertainties and the overall reliability of the measurements.

## Figures and Tables

**Table 1:** Statistical parameters the median (Med), the first and third quartiles (Q1, Q3), the Interquartile Range (IQR), and the distance of the first quartiles of fresh leaves and the third quartiles of dry leaves when the effect of the nominal wavelength as an uncertainty source on the NDVI values for different leaf states (LSs) and atmospheric conditions (ATM. Conds.) is evaluated.

LS	Atm. Cond.	Med	Q1	Q3	IQR	$Q1_{F}-Q3_{D}$
Fresh	Humid–Hazy	0.829	0.817	0.836	0.019	0.049
	Dry–Clear	0.799	0.785	0.813	0.028	0.054
	Mixed	0.812	0.799	0.824	0.025	0.049
Dry	Humid–Hazy	0.750	0.742	0.768	0.026	0.049
	Dry–Clear	0.719	0.703	0.731	0.028	0.054
	Mixed	0.733	0.714	0.750	0.036	0.049

**Table 2:** Statistical parameters the median (Med), the first and third quartiles (Q1, Q3), the Interquartile Range (IQR), and the distance of the first quartiles of fresh leaves and third quartiles of dry leaves when the effect of solar irradiance standard deviation as an uncertainty source on the NDVI values for different leaf states (LSs) and atmospheric conditions (ATM. Conds.) is evaluated.

LS	Atm. Cond.	Med	Q1	Q3	IQR	$Q1_{F}-Q3_{D}$
Fresh	Humid–Hazy	0.836	0.836	0.838	0.002	0.068
	Dry–Clear	0.797	0.797	0.798	0.001	0.076
	Mixed	0.809	0.797	0.836	0.039	0.029
Dry	Humid–Hazy	0.768	0.750	0.768	0.018	0.068
	Dry–Clear	0.721	0.719	0.721	0.002	0.076
	Mixed	0.737	0.721	0.768	0.047	0.029

**Table 3:** Statistical parameters the median (Med), the first and third quartiles (Q1, Q3), the Interquartile Range (IQR), and the distance of the first quartiles of fresh leaves and the third quartiles of dry leave when the effect of the camera SNR at 40 dB, 50 dB, and 60 dB as an uncertainty source on the NDVI values for different leaf states (LSs) and atmospheric conditions (ATM. Conds.) is evaluated.

LS	Atm. Cond.	SNR [dB]	Med	Q1	Q3	IQR	$Q1_{F}-Q3_{D}$
Fresh	Humid–Hazy	40	0.836	0.836	0.836	0.000	0.068
		50	0.836	0.836	0.836	0.000	0.068
		60	0.836	0.836	0.836	0.000	0.068
	Dry–Clear	40	0.797	0.797	0.797	0.000	0.076
		50	0.797	0.797	0.797	0.000	0.076
		60	0.797	0.797	0.797	0.000	0.076
	Mixed	40	0.817	0.797	0.836	0.039	0.029
		50	0.817	0.797	0.836	0.039	0.029
		60	0.817	0.797	0.836	0.039	0.029
Dry	Humid–Hazy	40	0.768	0.750	0.768	0.018	0.068
		50	0.768	0.768	0.768	0.000	0.068
		60	0.768	0.768	0.768	0.000	0.068
	Dry–Clear	40	0.721	0.721	0.721	0.000	0.076
		50	0.721	0.721	0.721	0.000	0.076
		60	0.721	0.721	0.721	0.000	0.076
	Mixed	40	0.735	0.721	0.768	0.047	0.029
		50	0.745	0.721	0.768	0.047	0.029
		60	0.745	0.721	0.768	0.047	0.029

**Table 4:** Statistical parameters the median (Med), the first and third quartiles (Q1, Q3), the Interquartile Range (IQR), and the distance of the first quartiles of fresh leaves and the third quartiles of dry leaves when the effect of the variability of the NDVI values as an uncertainty source on the NDVI values for different leaf states (LSs) and atmospheric conditions (ATM. Conds.) is evaluated.

No./LS	Atm. Cond.	Med	Q1	Q3	IQR	$Q1_{F}-Q3_{D}$
49/Fresh Leaf	Humid–Hazy	0.843	0.830	0.854	0.024	0.042
	Dry–Clear	0.818	0.802	0.826	0.024	0.057
	Mixed	0.827	0.813	0.845	0.032	0.040
49/Dry Leaf	Humid–Hazy	0.770	0.758	0.788	0.030	0.042
	Dry–Clear	0.729	0.714	0.745	0.031	0.057
	Mixed	0.752	0.727	0.773	0.046	0.040

**Table 5:** Statistical parameters the median (Med), the first and third quartiles (Q1, Q3), the Interquartile Range (IQR), and the distance of the first quartiles of fresh leaves and the third quartiles of dry leaves when the effect of the combination of all uncertainty sources on the NDVI values for different leaf states (LSs) and atmospheric conditions (ATM. Conds.) is evaluated.

No./LS	Atm. Cond.	SNR [dB]	Med	Q1	Q3	IQR	$Q1_{F}-Q3_{D}$
49/Fresh	Humid–Hazy	40	0.840	0.825	0.856	0.031	0.035
		50	0.842	0.822	0.854	0.032	0.039
		60	0.843	0.820	0.851	0.031	0.040
	Dry–Clear	40	0.815	0.796	0.831	0.035	0.036
		50	0.819	0.791	0.829	0.038	0.041
		60	0.813	0.797	0.832	0.035	0.047
	Mixed	40	0.829	0.808	0.843	0.035	0.038
		50	0.825	0.800	0.843	0.043	0.030
		60	0.828	0.810	0.842	0.032	0.040
49/Dry	Humid–Hazy	40	0.762	0.739	0.792	0.053	0.035
		50	0.767	0.739	0.783	0.044	0.039
		60	0.767	0.742	0.782	0.040	0.040
	Dry–Clear	40	0.737	0.704	0.755	0.051	0.036
		50	0.730	0.697	0.750	0.053	0.041
		60	0.731	0.699	0.751	0.052	0.047
	Mixed	40	0.743	0.724	0.768	0.044	0.038
		50	0.748	0.720	0.771	0.051	0.030
		60	0.748	0.718	0.770	0.052	0.040

**Table 6:** Statistical parameters the median (Med), the first and third quartiles (Q1, Q3), the Interquartile Range (IQR), and the distance of the first quartiles of fresh leaves and the third quartiles of dry leaves when the effect of the combination of all uncertainty sources by using the AQ600 camera’s features on the NDVI values for different leaf states (LSs) and atmospheric conditions (ATM. Conds.) is evaluated.

No./LS	Atm. Cond.	SNR [dB]	Med	Q1	Q3	IQR	$Q1_{F}-Q3_{D}$
49/Fresh	Humid–Hazy	40	0.842	0.824	0.857	0.033	0.041
		50	0.838	0.827	0.850	0.023	0.044
		60	0.840	0.824	0.852	0.028	0.047
	Dry–Clear	40	0.821	0.802	0.834	0.032	0.047
		50	0.819	0.797	0.831	0.034	0.050
		60	0.819	0.802	0.833	0.031	0.045
	Mixed	40	0.829	0.809	0.847	0.038	0.037
		50	0.827	0.806	0.843	0.037	0.034
		60	0.831	0.814	0.841	0.027	0.048
49/Dry	Humid–Hazy	40	0.763	0.741	0.783	0.042	0.041
		50	0.760	0.739	0.783	0.044	0.044
		60	0.763	0.748	0.777	0.029	0.047
	Dry–Clear	40	0.728	0.710	0.755	0.045	0.047
		50	0.729	0.704	0.747	0.043	0.050
		60	0.733	0.706	0.757	0.051	0.045
	Mixed	40	0.752	0.722	0.772	0.05	0.037
		50	0.745	0.713	0.772	0.059	0.034
		60	0.751	0.725	0.766	0.041	0.048

## Data Availability

Data are contained within the article.
